# Mal-angulation of femoral rotational osteotomies causes more postoperative sagittal mechanical leg axis deviation in supracondylar than in subtrochanteric procedures

**DOI:** 10.1186/s40634-020-00262-6

**Published:** 2020-07-01

**Authors:** Lukas Jud, Octavian Andronic, Lazaros Vlachopoulos, Sandro F. Fucentese, Patrick O. Zingg

**Affiliations:** Department of Orthopedics, Balgrist University Hospital, University of Zurich, Forchstrasse 340, 8008 Zürich, Switzerland

**Keywords:** Subtrochanteric osteotomy, Supracondylar osteotomy, Sagittal leg axis, Sagittal femoral bowing, Total knee arthroplasty, Intramedullary nailing

## Abstract

**Purpose:**

Alteration of the postoperative frontal mechanical leg axis is a known problem in femoral rotational osteotomies. However, the maintenance of the sagittal mechanical leg axis seems also important. Goal of this study was to investigate the impact of femoral rotational osteotomies on the sagittal mechanical leg axis and to identify the degree of mal-angulation of the osteotomy planes that alter the postoperative sagittal alignment relevantly.

**Methods:**

Using 3D bone models of two patients with a pathologic femoral torsion (42° antetorsion and 6° retrotorsion), subtrochanteric and supracondylar rotational osteotomies were simulated first with an osteotomy plane perpendicular to the mechanical femoral axis (baseline osteotomy plane), second with predefined mal-angulated osteotomy planes. Subsequently, five different degrees of rotation were applied and the postoperative deviations of the sagittal mechanical leg axes were analyzed.

**Results:**

Using the baseline osteotomy plane, the sagittal mechanical leg axis changed by 0.4° ± 0.5° over both models. Using the mal-angulated osteotomy planes, maximum deviation of the sagittal mechanical leg axis of 4.0° ± 1.2° and 11.0° ± 2.0° was observed for subtrochanteric and for supracondylar procedures, respectively. Relevant changes of more than 2° were already observed with mal-angulation of 10° in the frontal plane and 15° of rotation in supracondylar procedures.

**Conclusion:**

Relevant changes of the postoperative sagittal mechanical leg axis could be observed with just slight mal-angulation of the osteotomy planes, in particular in supracondylar procedures and in cases with higher degrees of rotation. However, osteotomies perpendicular to the femoral mechanical axis showed no relevant alterations.

## Background

Subtrochanteric or supracondylar femoral rotational osteotomies are established surgical treatment options in symptomatic patients with pathological increased femoral antetorsion or retrotorsion [1, 2]. However, by performing such reconstructive procedures, potential undesired changes are already known, in particular changes of the antero-posterior (AP)-projected mechanical leg axis [3–5]. Although corrective procedures of complex deformities should not only consider a single two-dimensional (2D) projection (i.e. AP-projection), but the whole three-dimensional (3D) anatomy, the sagittal plane did not receive much attention in the current literature so far. Lee et al. [6] showed in a computer model an extension of the femur in the sagittal plane by performing an intertrochanteric femoral rotational osteotomy with the osteotomy plane perpendicular to the anatomical axis of the proximal femur. This seems important, as changes of the sagittal femoral bowing are known to affect later surgical procedures such as total knee arthroplasty (TKA) [7, 8] or intramedullary nailing in proximal femoral fractures [9, 10]. It seems obvious, that reconstructive procedures should not be performed by accepting secondary problems and hamper later required surgical procedures in life. Therefore, it was the objective of this study to investigate postoperative sagittal mechanical leg axis deviations in subtrochanteric and supracondylar femoral rotational osteotomies. By using a computer simulation approach, first, subtrochanteric and supracondylar femoral rotational osteotomies were simulated perpendicular to the femoral mechanical axis [4] and with different degrees of rotation with consecutive measurement of the changes in the sagittal mechanical leg axis. Second, due to the difficult nature of such procedures and the possibility of unintended intraoperative mal-angulation of the osteotomy planes, the simulations and measurements were also performed with different degrees of mal-angulation of the osteotomy planes. Since hitherto no reference values for relevant sagittal mechanical leg axis deviations exist, reference values from the AP-projected mechanical leg axis were applied. Therefore, as mechanical leg axis correction in the AP-view shows accuracy of approximately 2° [11], it was likewise the objective of this study to identify the degree of mal-angulation of the osteotomy planes that alter the postoperative sagittal mechanical leg axis relevantly by more than 2°. The hypothesis is, that in femoral rotational osteotomies, an osteotomy perpendicular to the femoral mechanical axis should not alter the postoperative sagittal mechanical leg axis relevantly, but a mal-angulation of the osteotomy plane can result in relevant sagittal mechanical leg axis deviations.

## Methods

**Fig. 1:**
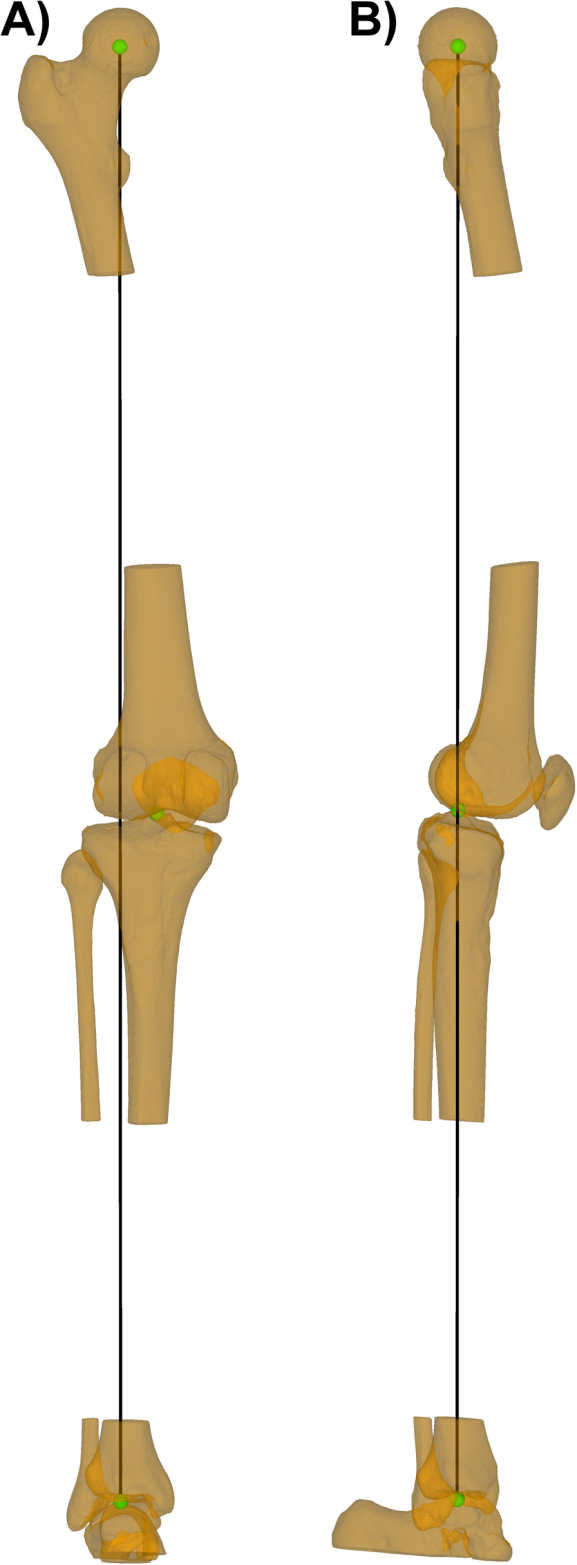
Alignment of the bone models. In green the hip-, knee-, and ankle-center. In black the axis connecting the hip- and ankle-center. **a** The bone model is oriented in an antero-posterior (AP) view. **b** The bone model is internally rotated by 90° to obtain a lateral view. The knee was controlled for full extension in a way that the plumb line from the hip- to the ankle-center passes exactly through the knee-center

**Fig. 2:**
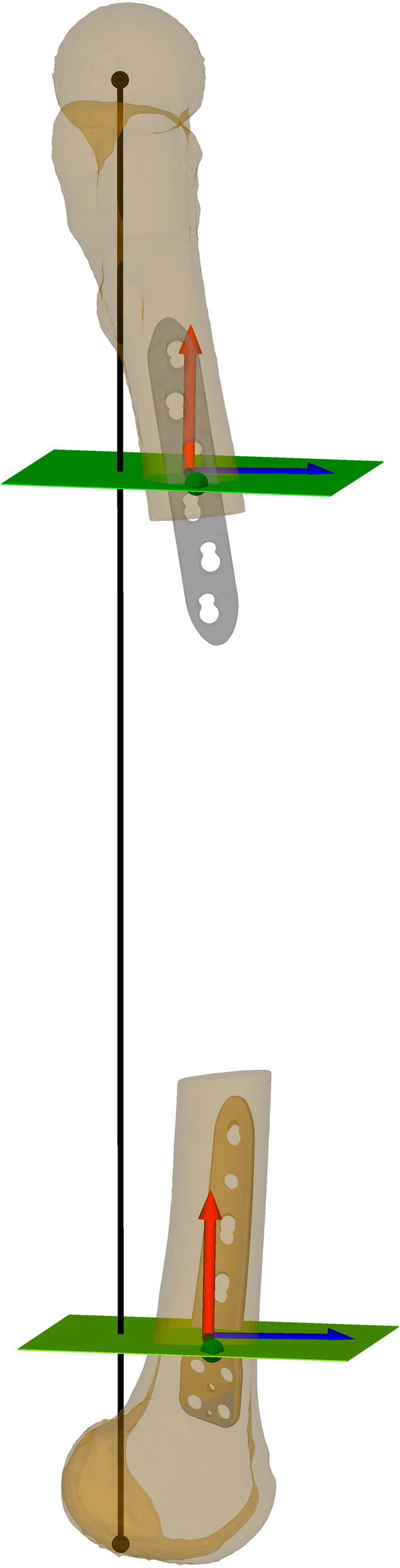
Baseline osteotomy planes. In black the mechanical femoral axis. In green the baseline osteotomy planes, defined perpendicular to the mechanical femoral axis and positioned in a way that the respective fixation plates (grey) could be properly placed. Standardized coordinate systems in the center of the osteotomy planes

**Fig. 3:**
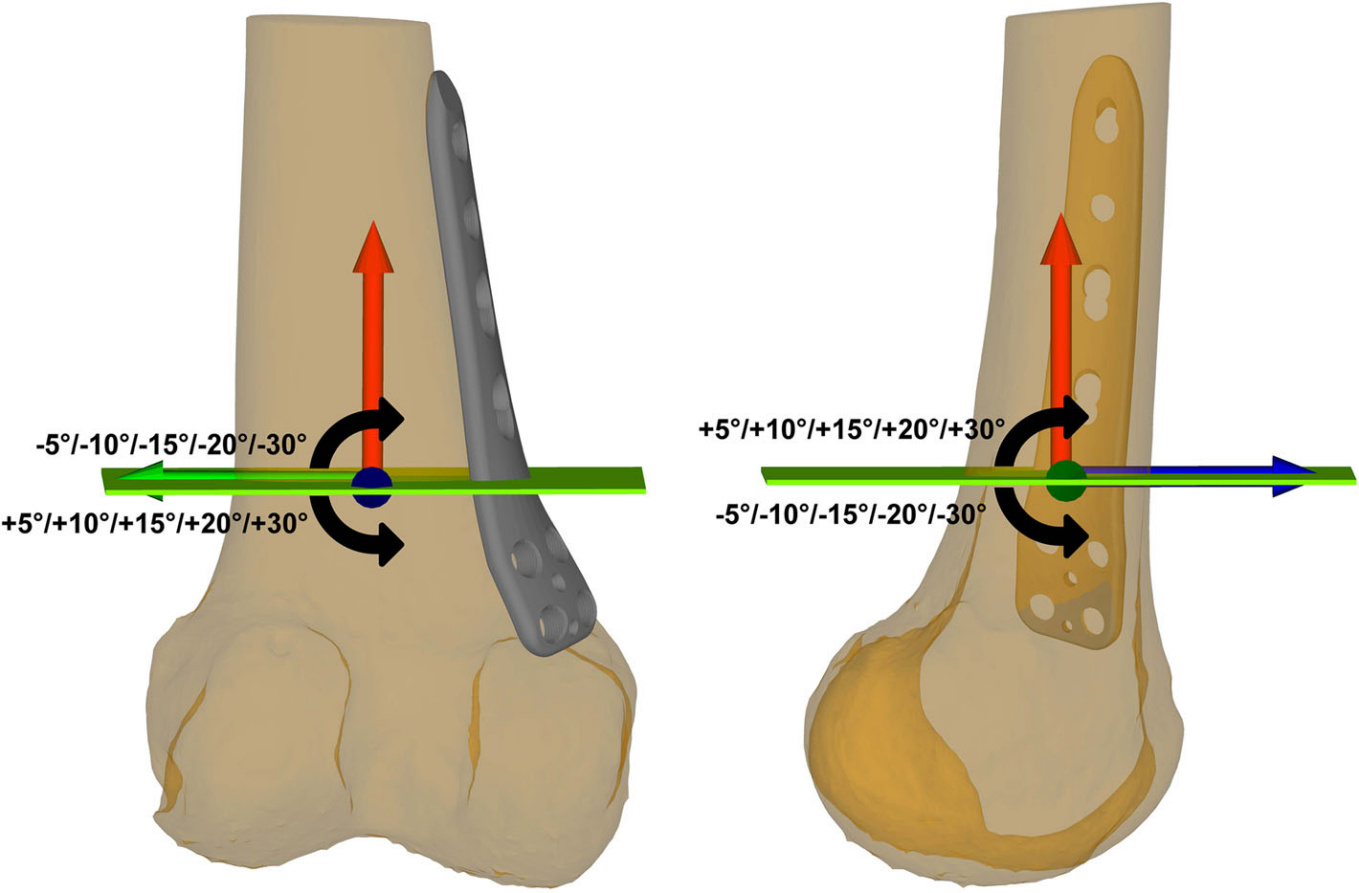
Mal-angulation of the osteotomy planes. Mal-angulation of the baseline osteotomy plane was performed along the standardized coordinate systems in the frontal plane around the x-axis (blue) and in the sagittal plane around the z-axis (green) in steps of +/− 5°, +/− 10°, +/− 15°, +/− 20°, +/− 30

**Fig. 4:**
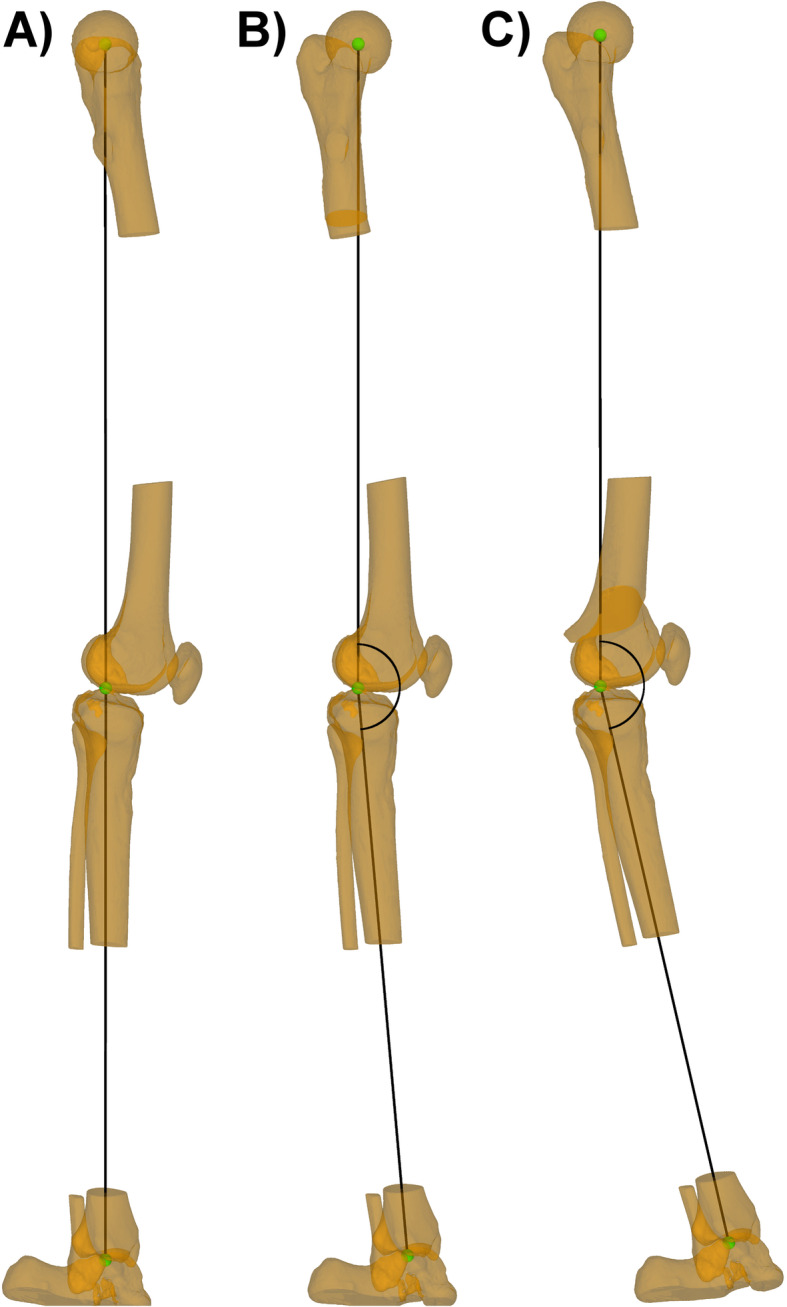
Measurement of the sagittal mechanical leg axis. **a** Starting position with a neutral alignment. **b** 30° of internal rotation in Model 2 after osteotomy with a mal-angulation of the subtrochanteric osteotomy plane of + 30° in the frontal plane. **c** 30° of internal rotation in Model 2 after osteotomy with a mal-angulation of the supracondylar osteotomy plane of + 30° in the frontal plane. Schematically marked is the measurement of the altered sagittal mechanical leg axis

Computed tomography (CT) data of the lower extremity of two patients with a femoral rotational deformity was used, whereby the CT data of both patients was already used in a different study [4]. The first patient showed an increased femoral antetorsion (42 degrees of antetorsion, Model 1), the second patient showed a reduced femoral antetorsion (6 degrees of retrotorsion, Model 2). The mechanical leg axis, measured as the hip-knee-ankle angle, showed to be 2.4° valgus in Model 1 and 5.1° valgus in Model 2. None of the patients were suffering from a posttraumatic deformity and except the rotational deformity, both patients had a normal femoral anatomy. Therefore, the mechanical lateral distal femoral angle (mLDFA) was 85° in Model 1 and 86° in Model 2 and the femoral antecurvatum angle was 8° and 14°, respectively. Segmentation of the CT data was performed using commercial segmentation software (Mimics Medical 19.0, Materialise NV, Leuven, Belgium) to create 3D surface models of the lower extremities of the two patient models. In a next step, the bone models were imported into the in-house developed surgical planning software CASPA (Balgrist CARD AG, Zurich, Switzerland). The bone models were oriented in a same way as needed for the measurement of the AP-projected 3D mechanical leg axis [12]. To correct a slight flexion position during CT acquisition, the bone models were controlled for full extension using a same method as described by Jud et al. [13]. Hereby, a neutral alignment of the sagittal mechanical leg axis of 0° is achieved. Afterwards, the bone models were internally rotated by 90° around the axis connecting the hip- and ankle-center to obtain a lateral view of the lower extremity (Fig. 1). The mechanical femoral axis was implemented from the femoral head center to the intercondylar notch center. Subsequently, the baseline osteotomy planes (i.e. subtrochanteric and supracondylar osteotomy plane) were defined perpendicular to the mechanical femoral axis [4] and in reconciliation that a 6 holes 4.5 mm Broad LCP Plate (Depuy-Synthes Oberdorf, Switzerland), respectively a TomoFix Medial Distal Femur Plate (Depuy-Synthes Oberdorf, Switzerland) could be correctly placed (Fig. 2). For mal-angulation of the baseline osteotomy planes, a standardized coordinate system was defined in the center of the respective osteotomy planes. The centers were determined as the center of the mass of a 1 mm slice of the bones at the level of the respective baseline osteotomies. The axes of the standardized coordinate system were oriented in accordance to the International Society of Biomechanics (ISB) recommendation on definitions of joint coordinate systems [14] and the z-axes of the respective coordinate systems were further oriented in accordance to [4] (Fig. 2). Subsequently, subtrochanteric and supracondylar osteotomies were performed in both patient models with the baseline osteotomy planes. Afterwards, the baseline osteotomy planes were successively mal-angulated with respect to the sagittal plane (z-axis) and the frontal plane (x-axis), according to the standardized coordinate systems. The steps of mal-angulation were defined to be +/− 5°, +/− 10°, +/− 15°, +/− 20°, and +/− 30° (Fig. 3). Due to the increased femoral antetorsion in Model 1, after the osteotomy with the baseline and all mal-angulated osteotomy planes, external rotation of the bones distal from the osteotomy was performed by 5°, 10°, 15°, 20°, and 30° around the y-axis (axial plane) of the standardized coordinate system. In Model 2, internal rotation of the distal bones was performed due to the decreased femoral antetorsion, respectively due to the femoral retrotorsion, using the same steps of rotation. In both models, after each osteotomy with every step of rotation, the sagittal mechanical leg axis was measured using the hip-, knee-, and ankle-center, in a same way like the AP-projected 3D mechanical leg axis is measured [12], but using the lateral view instead of the AP view (Fig. 4). For interpretation of the data, absolute values of deviations of the sagittal mechanical leg axis were used.

### Statistical analysis

The statistical analysis was performed by descriptive analysis using the software R (version 1.1.463; R foundation, Vienna, Austria).

## Results

**Fig. 5:**
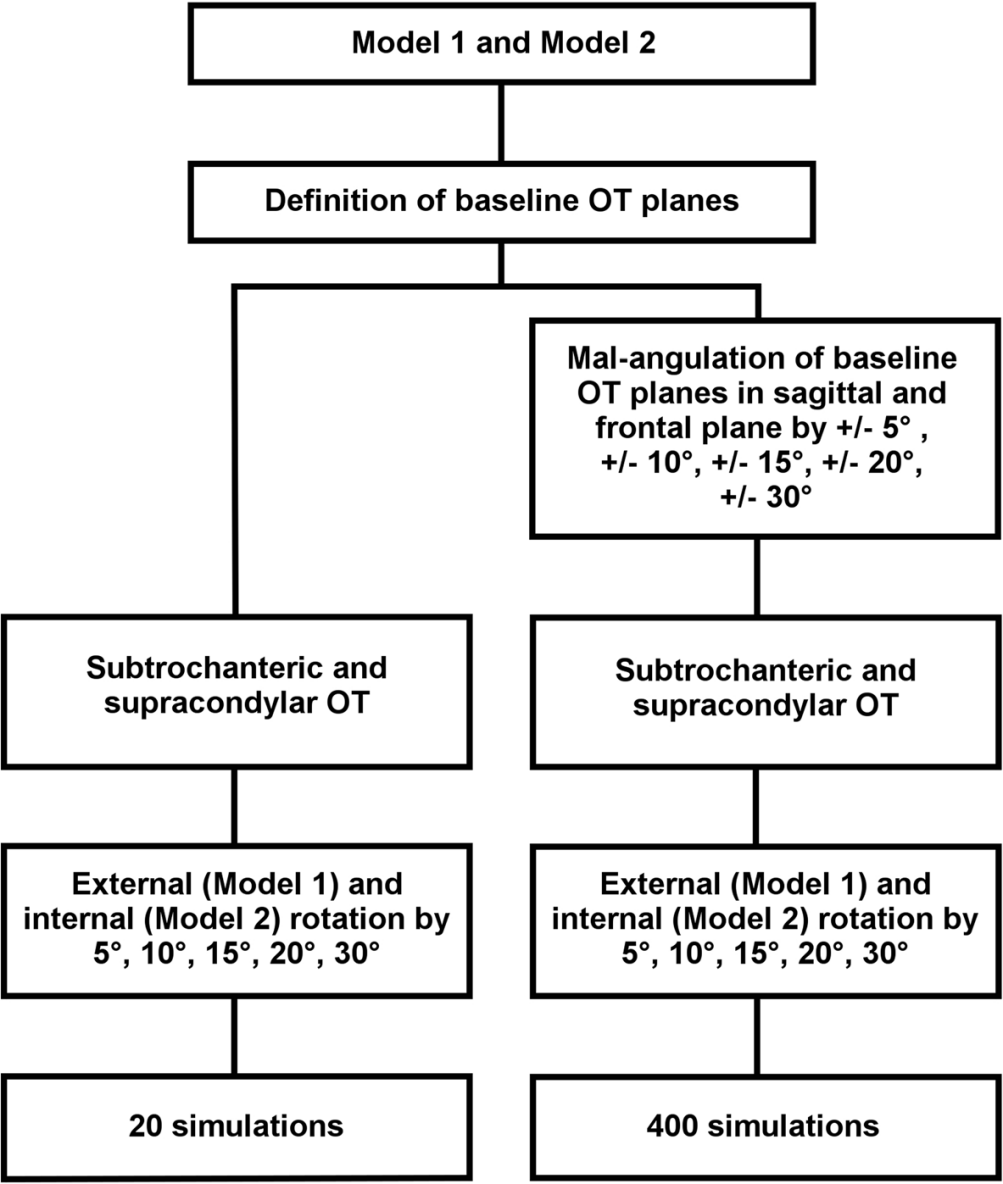
Flowchart of the performed simulations. OT: Osteotomy

Over both models, 20 rotational osteotomies with different degrees of rotation were performed using the baseline osteotomy planes. Regarding the mal-angulated osteotomies, 80 different mal-angulated osteotomy planes have been generated and with the five different degrees of rotation, overall 400 simulations with mal-angulated osteotomy planes were performed in the two patient models (Fig. 5).

Using the baseline osteotomy planes, the simulations with the different degrees of rotation altered the neutral sagittal alignment by a mean of 0.4° ± 0.5°. In Model 1, the postoperative mean sagittal mechanical leg axis was 0.4° ± 0.5° and in Model 2 0.4° ± 0.4°, compared to the preoperative neutral alignment of 0°.

Regarding the simulated rotational osteotomies using the mal-angulated osteotomy planes, an overview of mean deviation from preoperative sagittal mechanical leg axis for the subtrochanteric osteotomies is given in Table 1, and an overview for the supracondylar osteotomies is given in Table 2. In supracondylar procedures, higher degrees of rotation and higher degrees of mal-angulation of the osteotomy plane always resulted in higher degrees of mean postoperative sagittal mechanical leg axis deviations. In subtrochanteric procedures, likewise higher degrees of rotation and higher degrees of mal-angulation of the osteotomy plane in the frontal plane always resulted in higher degrees of mean postoperative sagittal mechanical leg axis deviations. With increasing mal-angulation of the osteotomy plane in the sagittal plane for subtrochanteric procedures, only a slight decrease of mean postoperative sagittal mechanical leg axis deviations could be observed.

**Table 1:** Absolute mean deviations from the preoperative sagittal mechanical leg axis in subtrochanteric rotational osteotomies

Angulation Error of the Osteotomy Plane	Plane	Error in the Sagittal Leg Axis Alignment per Rotation				
		5°	10°	15°	20°	30°
+/− 5°	Sagittal	0.4° ± 0.3°	0.6° ± 0.3°	0.9° ± 0.3°	1.1° ± 0.3°	1.6° ± 0.4°
	Frontal	0.2° ± 0.1°	0.5° ± 0.3°	0.7° ± 0.4°	1.0° ± 0.5°	**1.4° ± 0.7°**
+/− 10°	Sagittal	0.2° ± 0.1°	0.5° ± 0.1°	0.7° ± 0.1°	0.9° ± 0.1°	1.4° ± 0.2°
	Frontal	0.3° ± 0.2°	0.5° ± 0.4°	0.7° ± 0.7°	1.0° ± 0.9°	**1.4° ± 1.4°**
+/− 15°	Sagittal	0.2° ± 0.1°	0.5° ± 0.1°	0.7° ± 0.1°	0.9° ± 0.1°	1.4° ± 0.3°
	Frontal	0.4° ± 0.2°	0.7° ± 0.5°	1.0° ± 0.7°	**1.4° ± 0.9°**	**2.1° ± 1.4°**
+/− 20°	Sagittal	0.2° ± 0.1°	0.4° ± 0.1°	0.7° ± 0.1°	0.9° ± 0.1°	1.3° ± 0.4°
	Frontal	0.5° ± 0.2°	0.9° ± 0.4°	**1.4° ± 0.7°**	**1.8° ± 0.9°**	**2.7° ± 1.3°**
+/− 30°	Sagittal	0.2° ± 0.1°	0.4° ± 0.1°	0.6° ± 0.1°	0.8° ± 0.2°	1.2° ± 0.5°
	Frontal	0.7° ± 0.2°	1.3° ± 0.4°	**2.0° ± 0.6°**	**2.7° ± 0.8°**	**4.0° ± 1.2°**

Deviations greater than 2° (mean value plus standard deviation) have been marked bold.

**Table 2:** Absolute mean deviations from the preoperative sagittal mechanical leg axis in supracondylar rotational osteotomies

Angulation Error of the Osteotomy Plane	Plane	Error in the Sagittal Leg Axis Alignment per Rotation				
		5°	10°	15°	20°	30°
+/− 5°	Sagittal	0.1° ± 0.1°	0.3° ± 0.2°	0.4° ± 0.3°	0.6° ± 0.4°	1.0° ± 0.6°
	Frontal	0.4° ± 0.0°	0.7° ± 0.1°	1.0° ± 0.2°	1.3° ± 0.2°	**2.1° ± 0.7°**
+/− 10°	Sagittal	0.2° ± 0.2°	0.5° ± 0.4°	0.8° ± 0.7°	1.1° ± 0.9°	**2.0° ± 1.2°**
	Frontal	0.7° ± 0.1°	1.4° ± 0.2°	**2.0° ± 0.3°**	**2.6° ± 0.4°**	**3.7° ± 0.8°**
+/− 15°	Sagittal	0.4° ± 0.3°	0.7° ± 0.7°	**1.2° ± 1.0°**	**1.7° ± 1.3°**	**3.0° ± 1.9°**
	Frontal	1.0° ± 0.1°	**2.0° ± 0.3°**	**3.0° ± 0.4°**	**3.9° ± 0.6°**	**5.6° ± 1.1°**
+/− 20°	Sagittal	0.5° ± 0.3°	1.0° ± 0.8°	**1.6° ± 1.2°**	**2.3° ± 1.6°**	**4.1° ± 2.3°**
	Frontal	1.4° ± 0.2°	**2.7° ± 0.3°**	**4.0° ± 0.6°**	**5.2° ± 0.8°**	**7.4° ± 1.4°**
+/− 30°	Sagittal	0.7° ± 0.6°	**1.3° ± 1.3°**	**2.2° ± 1.9°**	**3.2° ± 2.5°**	**5.6° ± 3.7°**
	Frontal	**2.0° ± 0.2°**	**3.9° ± 0.5°**	**5.8° ± 0.8°**	**7.6° ± 1.2°**	**11.0° ± 2.0°**

Deviations greater than 2° (mean value plus standard deviation) have been marked bold.

## Discussion

**Fig. 6:**
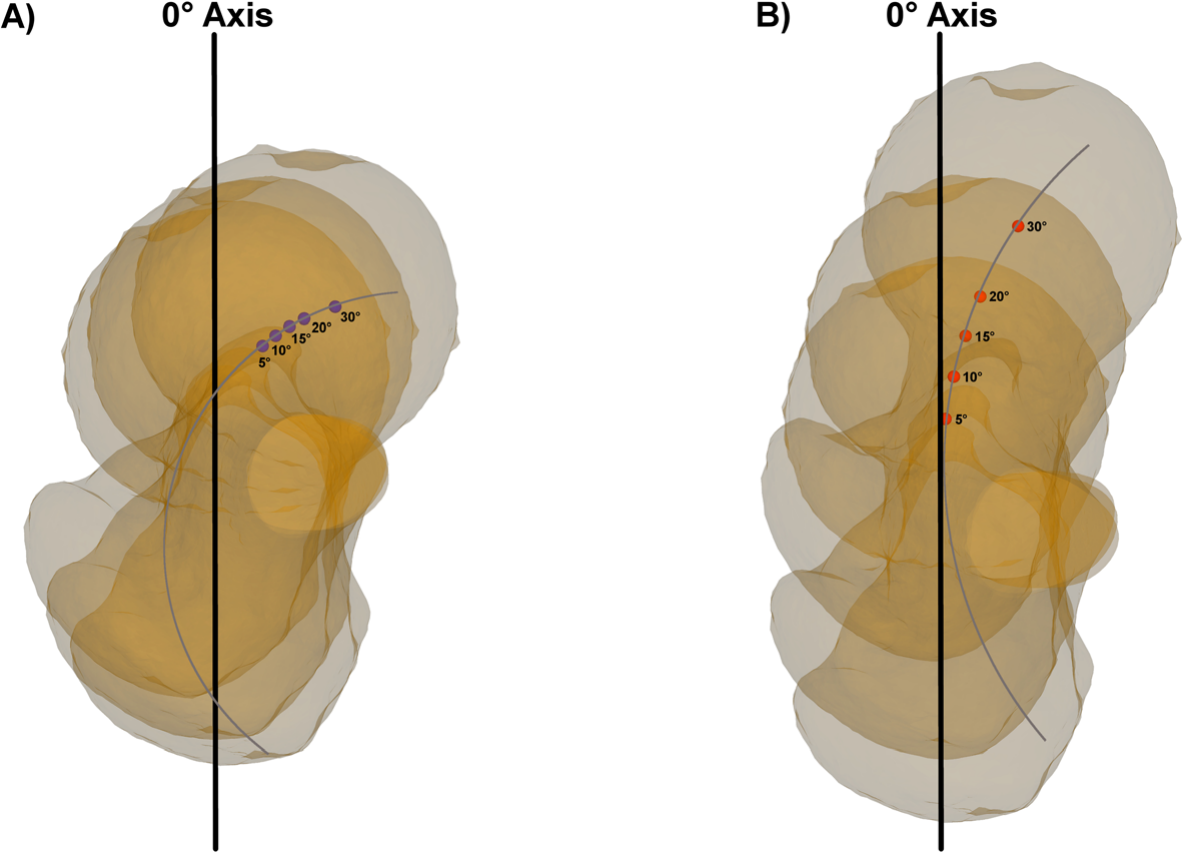
Effect of decreased postoperative sagittal mechanical leg axis deviation with increased mal-angulation in subtrochanteric procedures. **a** Subtrochanteric rotational osteotomy in Model 2 using an osteotomy plane with + 5° sagittal mal-angulation. Marked is the sagittal projected 0° axis in black and the femoral head centers for every step of rotation in purple. In grey schematically marked is the relative circle of movement of the femoral head center. **b** Subtrochanteric rotational osteotomy using an osteotomy plane with + 30° sagittal mal-angulation. In red the femoral head centers for ever step of rotation. Compared to Fig. 6a, the relative circle of movement of the femoral head center is flatter and more in line with the 0° axis

The most important finding of this study is, that performing a femoral rotational osteotomy perpendicular to the mechanical femoral axis showed no relevant postoperative sagittal mechanical leg axis deviation. However, performing the osteotomy with an unintended mal-angulated osteotomy plane showed mean deviations of the postoperative sagittal mechanical leg axis up to 11.0° ± 2.0°, wherefore the hypothesis of this study could be confirmed. In supracondylar procedures, even a mal-angulation of only 5° in the frontal plane in combination with a rotation of 30°, or a mal-angulation of 10° in the frontal plane in combination with a rotation of only 15° resulted in a postoperative mean deviation of the sagittal mechanical leg axis greater than 2°. Overall, the findings of this study reveal, that supracondylar procedures showed to be more vulnerable for relevant postoperative sagittal mechanical leg axis deviations than subtrochanteric procedures, the same applies for mal-angulation of the osteotomy plane in the frontal plane compared to mal-angulation in the sagittal plane. The difference of the alteration of the postoperative sagittal mechanical leg axis between subtrochanteric and supracondylar procedures can be explained according to the described tendency to varus angulation in proximal femoral rotational osteotomies and the tendency to valgus angulation in distal femoral rotational osteotomies [5]. While proximal procedures more affect the AP-projected relative femoral neck length, with the applied degrees of rotation, the generated deviation mainly gets projected into the AP-view. In distal procedures, due to the proximity of the center of rotation of the respective osteotomy to the mechanical femoral axis, the generated deviation gets earlier projected into the sagittal-view. Furthermore, in subtrochanteric procedures, a slight decrease of the postoperative mean sagittal mechanical leg axis deviations could be observed with increased mal-angulation of the osteotomy plane in the sagittal plane. The relative circle of movement of the femoral head center during rotation can explain this circumstance. With increased mal-angulation in the sagittal plane, the relative circle of movement of the femoral head center gets more in line with the sagittal projected neutral sagittal mechanical leg axis in subtrochanteric procedures, wherefore the mean deviation of the postoperative sagittal mechanical leg axis decreased (Fig. 6). However, in return, the postoperative deviation with mal-angulation of the osteotomy plane in the sagittal plane adds up in the AP-projected mechanical leg axis [4]. In all other simulations, the relative circle of movement of the femoral head center moved more notable away from the neutral sagittal mechanical leg axis with increased mal-angulation, wherefore increased postoperative deviations of the sagittal mechanical leg axis could be observed.

Overall, the observation of relevant sagittal mechanical leg axis deviations in femoral rotational osteotomies, already with just slight mal-angulation of the osteotomy planes, appear important, as not only the maintenance of the AP-projected mechanical leg axis but also the maintenance of the sagittal mechanical leg axis seems crucial in such procedures. With a continuously increasing incidence of knee osteoarthritis, also a continuously increase of TKA can be expected [15]. However, it could be shown that in patients undergoing TKA, alterations of the sagittal femoral bowing affect the femoral implant position, possibly resulting in a flexion or extension position of the implant [7, 8]. It has been shown, that a femoral implant position in a flexion position potentially increases anterior tibial post impingement and changes the flexion gap configuration, whereas an extension position results in anterior cortex notching [16–18]. While the risk for a supracondylar femoral fracture in anterior cortex notching is controversially discussed [17, 19, 20], its relevance for the implant longevity is not yet clear. However, a flexed position of the femoral implant with an anterior tibial post impingement potentially results in early loosening of the implant and therefore likely affects the implant longevity [16, 18, 21]. Another obstacle in patients with an altered sagittal femoral bowing, can be the need of intramedullary nailing in case of proximal femoral fracture, that is estimated to occur in 238′000 hips annually in the United States and the number is expected to increase continuously [22]. Due to the rigid nature of the standard femoral intramedullary nail designs, a mismatch between the altered sagittal femoral bowing and the curvature radius of the nail can be a problem and potentially results in cortical perforation or angulation of the fracture [9, 10, 23].

It seems obvious, that reconstructive procedures, such as femoral rotational osteotomies, should not be performed in a way that later required surgical procedures get hampered. An accurate preoperative planning seems mandatory. However, using conventional surgical techniques, an intraoperative estimation of the femoral mechanical axis is challenging due to the limited surgical exposure. Even more challenging is to perform the osteotomy plane exactly perpendicular to the femoral mechanical axis, and therefore the risk for an unintended mal-angulated osteotomy plane with a possible related sagittal mechanical leg axis deviation increases. Hence, the use of navigation aids, such as patient-specific instruments (PSI) [24], should probably be considered in such surgical procedures to maintain the sagittal mechanical leg axis. In particular this applies to supracondylar procedures and in cases with higher degrees of intended rotation, as it has already been proposed to maintain the AP-projected mechanical leg axis [4].

This study has some limitations. First, only two patient models were used. However, with 42° femoral antetorsion in Model 1, and 6° femoral retrotorsion in Model 2, the two patient models presumably cover the deformities in daily practice. Likewise, this study was not thought to give an exact estimation of the expected postoperative deviation of the sagittal mechanical leg axis. The intent of this study was much more to demonstrate the presumably underestimated changes in the sagittal plane after femoral rotational osteotomies and to sensitize for this so far less investigated problem. Second limitation is the use of a computer simulation approach only. To perform a more comprehensive error analysis, cadaver experiments can be performed. However, the efforts and costs for cadaver experiments are very high and in contrast a computer simulation approach is a cost-effective and already established alternative. Third, the whole sagittal mechanical leg axis was investigated, not only the changes in the sagittal femoral bowing. The reason for this was, that a special developed CT protocol was used for preoperative planning, scanning only the regions of interest (i.e. proximal femur, distal femur, proximal tibia, distal tibia, distal fibula and the talus) and skipping irrelevant mid-shaft regions. Therefore, CT data of the whole femur was not available to measure explicitly the changes of the sagittal femoral bowing. However, by preoperatively orienting the bone models in a neutral sagittal position, the simulations reveal indirectly the changes of the sagittal femoral bowing. Fourth, as reference values for relevant postoperative sagittal mechanical leg axis deviations, reference values from the AP-projected mechanical leg axis were applied. Due to the fact, that so far no reference values for the sagittal mechanical leg axis exist, this circumstance had to be accepted, but is subject to future studies.

## Conclusion

Relevant changes of the postoperative sagittal mechanical leg axis could be observed in femoral rotational osteotomies with solely 5° of mal-angulation of the osteotomy planes. However, osteotomies perpendicular to the femoral mechanical axis showed no relevant alterations. To prevent problems in later required surgical treatments around the hip and knee, accurate preoperative planning is mandatory and surgical navigation aids should probably be considered. This applies in particular for supracondylar procedures and in cases with higher degrees of rotation.

## Data Availability

Anonymized source data can be obtained from the corresponding author on reasonable request.

## Abbreviations

AP: Antero-posterior
2D: Two-dimensional
3D: Three-dimensional
TKA: Total knee arthroplasty
CT: Computed tomography
mLDFA: Mechanical lateral distal femoral angle
ISB: International Society of Biomechanics
PSI: Patient-specific instruments
